# Long COVID and Type I IFN Signature in Working-Age Adults: A Cross-Sectional Study

**DOI:** 10.3390/ijms26189089

**Published:** 2025-09-18

**Authors:** Letizia Santinelli, Elio Gentilini Cacciola, Luca Bortolani, Marco Ridolfi, Luca Maddaloni, Federica Frasca, Matteo Fracella, Ginevra Bugani, Gabriella d’Ettorre, Claudio M. Mastroianni, Giancarlo Ceccarelli, Gabriele d’Ettorre

**Affiliations:** 1 Department of Public Health and Infectious Diseases, University of Rome Sapienza, 00185 Rome, Italy; letizia.santinelli@uniroma1.it (L.S.); luca.bortolani@uniroma1.it (L.B.); luca.maddaloni@uniroma1.it (L.M.); federica.frasca@uniroma1.it (F.F.); ginevra.bugani@uniroma1.it (G.B.); gabriella.dettorre@uniroma1.it (G.d.); claudio.mastroianni@uniroma1.it (C.M.M.); 2 UOC Malattie Infettive, Azienda Ospedaliera per l’emergenza, Cannizzaro, 95021 Catania, Italy; gentilini.cacciola.elio@gmail.com; 3 Department of Internal Medicine, Endocrine-Metabolic Sciences and Infectious Diseases, AOU Policlinico Umberto I, 00161 Rome, Italy; ma.ridolfi@policlinicoumberto1.it; 4 Department of Molecular Medicine, Laboratory of Virology, University of Rome Sapienza, 00185 Rome, Italy; matteo.fracella@uniroma1.it; 5 Local Health Authority ASL, 73100 Lecce, Italy; gabriele.det@libero.it

**Keywords:** long COVID, PASC, working-age people, IFN-I, ISG

## Abstract

To investigate relevant biomarkers that might aid in the diagnosis and monitoring of long COVID (LC), an analysis of IFN-α, IFN-β, ISG15, and ISG56 transcripts was performed by Real-Time PCR among people of working age who had been infected with SARS-CoV-2 one year prior to the study [LC and non-long COVID (NLC)]. Despite no differences in the transcript levels of IFN-α, IFN-β, ISG15, and ISG56 between LC and NLC, higher IFN-β mRNA levels were observed among LC compared to NLC individuals who were hospitalized for more than 10 days during acute SARS-CoV-2 infection. Moreover, previously SARS-CoV-2 infected participants that did not require respiratory support and developed LC exhibited higher levels of IFN-α and IFN-β compared to NLC with the same clinical characteristics. These results highlight that SARS-CoV-2 infection leads to changes in peripheral innate immune pathways, which could have implications for the development of LC.

## 1. Introduction

Data from recent literature indicate that the long-term effects following the post-acute phase of SARS-CoV-2 infection remain poorly understood and are still under active investigation [1,2]. However, a condition now widely referred to as “long COVID” (LC), or more formally “post-acute sequelae of SARS-CoV-2 infection” (PASC), has emerged as a significant public health concern. Long COVID is characterized by a spectrum of persistent symptoms, including fatigue, dyspnea, cognitive dysfunction, and neuropsychiatric disturbances, that can last for weeks or even months following apparent clinical and virological recovery [3,4]. From an immunological standpoint, it has been well established that SARS-CoV-2 is capable of evading innate immune detection by interfering with the molecular machinery involved in viral sensing. Among the multiple key factors associated with COVID-19, IFNs have drawn great attention, with specific emphasis on type I interferon (IFN-I), a multi-gene family of structurally similar cytokines, including IFN-α and IFN-β, which activates rapidly after sensing PAMPs and is constantly produced until the viral stimulus persists [5].

Therefore, IFN-I plays a central role in the innate immune response against viral infections, acting as the first line of defense by limiting viral replication and activating downstream immune pathways. In the context of SARS-CoV-2 infection, several studies have shown that a robust and timely IFN-I response is associated with milder disease, while delayed or suppressed IFN signaling correlates with severe COVID-19 outcomes [6,7].

SARS-CoV-2 has developed multiple strategies to evade this pathway, including direct inhibition of IFN production and signaling through viral proteins such as Open Reading Frame 6 (ORF6), ORF9b, and non-structural protein 1 (Nsp1); specifically, the virus targets upstream pattern recognition receptors and signaling molecules such as anti-melanoma differentiation-associated gene 5 (MDA5), retinoic acid-inducible gene-I (RIG-I), and mitochondrial antiviral signaling protein (MAVS) [6,8].

Furthermore, SARS-CoV-2 proteins have been shown to inhibit the downstream activity of IFN-stimulated genes (ISGs), thereby undermining the host’s antiviral response [9]. In fact, the SARS-CoV-2 main protease (M^pro^) significantly suppresses the expression and transcription of downstream ISGs driven by IFN-stimulated responses in a dose-dependent manner [10]. Moreover, during acute infection, patients with persistently high titers of anti-IFN-α neutralizing antibodies (NAB) showed a reduced expression of ISG15 and ISG56 transcripts, defined as surrogate markers of IFN bioactivity [11].

Recent evidence suggests that dysregulation of the IFN response may not only influence the acute phase but also contribute to the pathogenesis of LC. Persistent low-level inflammation, aberrant ISG expression, and immune exhaustion have been observed several months after acute infection, pointing to a possible failure in the resolution of the antiviral response [12,13]. Moreover, altered expression of ISGs has been reported in individuals with long-lasting symptoms, supporting the hypothesis that chronic IFN signaling dysregulation may underlie some aspects of LC, including fatigue, neurocognitive impairment, and autoimmunity [14]. Given the critical role of IFN-I in controlling viral infections, dysregulation of this pathway may contribute not only to acute disease severity but also to persistent post-viral inflammation. Therefore, understanding the dynamics of IFN responses in both the acute and post-acute phases of SARS-CoV-2 infection is critical for identifying biomarkers that can predict or monitor the development of long-term sequelae, as well as for pinpointing novel therapeutic strategies.

This work is driven by the urgent clinical need to elucidate the pathophysiological basis of LC and to identify molecular biomarkers that could support diagnosis and longitudinal monitoring of affected individuals [13,15]. In addition, considering that LC disproportionately affects individuals of working age—resulting in reduced quality of life, absenteeism, and impaired work performance—our study focused on this demographic segment [16,17].

By evaluating the expression profiles of genes involved in the IFN signaling pathway, we aim to shed light on possible mechanisms of immune dysregulation that may persist beyond the acute phase and contribute to the prolonged symptomatology observed in LC. This may also provide insights into targeted therapeutic interventions and rehabilitation strategies.

## 2. Results

### 2.1. Clinical and Demographic Characteristics of Study Population

Among the individuals hospitalized with SARS-CoV-2 from March 2020 to November 2021, 34 consented to participate and were enrolled. Of these, 26 individuals reporting two or more symptoms consistent with PASC were classified as LC, while the remaining 8 were categorized as non-long COVID (NLC). Demographic and clinical characteristics of the study population are reported in Table 1. The most common symptoms reported by LC are shown in Table 2.

### 2.2. IFN-I and ISGs Expression Among Previously SARS-CoV-2 Infected Individuals According to the Development of LC

**Figure 1:**
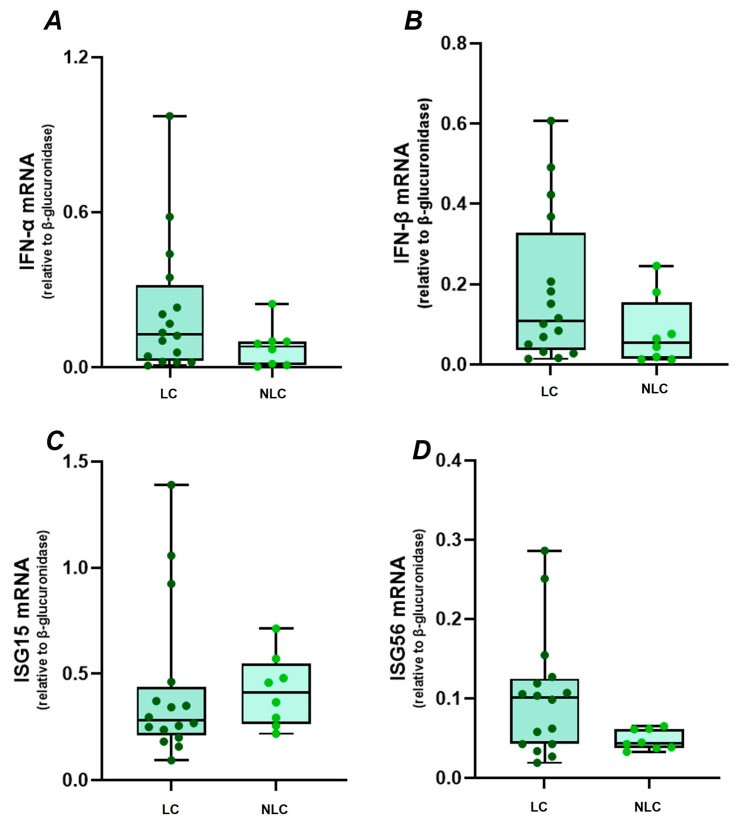
Gene expression levels of IFN-α (**A**), IFN-β (**B**), ISG15 (**C**), and ISG56 (**D**) in PBMC of LC (*n* = 16) and non-long COVID (NLC) (*n* = 8) individuals. Gene expression values between LC and NLC were compared by Mann–Whitney U-test.

Knowing that intensity of the immune response is a critical factor during COVID-19 [18], considering the clinical need to better comprehend the pathophysiological basis of LC, and with the aim to ascertain relevant biomarker(s) that might aid in the diagnosis and objective monitoring of the disease, we compared the transcript levels of IFN-α, IFN-β, ISG15 and ISG56 in PBMC from a subgroup of previously SARS-CoV-2 infected participants (*n* = 24), stratified as LC (*n* = 16) and NLC (*n* = 8). To avoid confounding factors that could interfere with the gene expression analysis, we tested plasma samples for the presence of anti-IFN-I NAB; all patients were negative for anti-IFN-α/β NAB (<10 TRU/mL). Similar levels of IFN-α (Figure 1A), IFN-β (Figure 1B), ISG15 (Figure 1C), and ISG56 (Figure 1D) were observed (*p* > 0.05) between LC and NLC.

The results of the multivariable linear regression models are summarized in Table 3. In the model predicting IFN-α mRNA expression and in the one for IFN-β mRNA, a higher Charlson Comorbidity Index (CCI ≥ 2) was significantly associated with reduced expression (adjusted β = −1.54 and −0.18, respectively; 95% CI: −2.80 to −0.27 and −0.29 to −0.07, respectively; *p* = 0.020 and 0.002, respectively). In contrast, the model predicting ISG56 mRNA expression showed multiple significant findings: LC status (adjusted β = 0.55; 95% CI: 0.11 to 0.99; *p* = 0.016) and hospitalization ≥ 10 days (adjusted β = 0.51; 95% CI: 0.08 to 0.94; *p* = 0.022) were both associated with increased expression levels, whereas a CCI ≥ 2 was associated with decreased ISG56 mRNA expression (adjusted β = −0.62; 95% CI: −1.18 to −0.05; *p* = 0.034).

### 2.3. Association Between Clinical Features of Acute Infection, LC, and IFN-I Response

**Figure 2:**
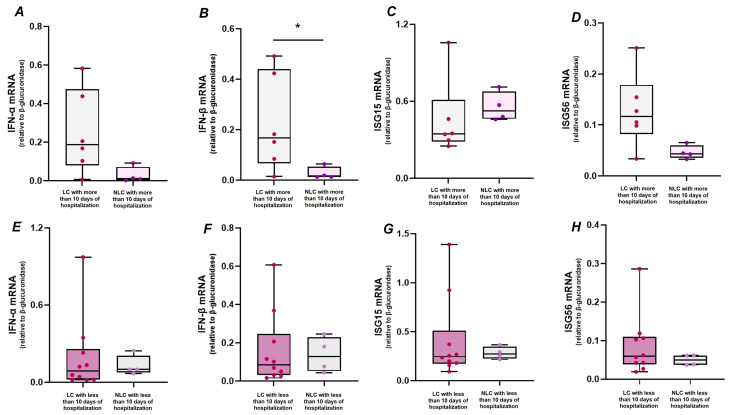
Gene expression levels of IFN-α (**A**,**E**), IFN-β (**B**,**F**), ISG15 (**C**,**G**), and ISG56 (**D**,**H**) in PBMC of LC with more than 10 days of hospitalization (*n* = 6), NLC with more than 10 days of hospitalization (*n* = 4), LC with less than 10 days of hospitalization (*n* = 10), NLC with less than 10 days of hospitalization (*n* = 4). Gene expression values were compared between LC and NLC with more than 10 days of hospitalization and between LC and NLC with less than 10 days of hospitalization by the Mann–Whitney U-test, * *p*  <  0.05.

Study participants were further stratified according to the days of hospitalization (<10 or >10 days) and the need for respiratory support (yes or no) during the acute infection, in order to identify any clinical features that might be associated with the expression of IFN-I and ISGs observed after one year. The results showed higher levels of IFN-β transcript in LC (*n* = 6) than NLC (*n* = 4) individuals who were hospitalized for more than 10 days (Figure 2B, *p* = 0.033), despite no significant differences were observed for IFN-α, ISG15 and ISG56, while comparison between LC (*n* = 10) and NLC (*n* = 4), both hospitalized for less than 10 days, revealed similar transcript levels of IFN-I (IFN-α and IFN-β) (Figure 2E,F), ISG15 (Figure 2G), and ISG56 (Figure 2H).

**Figure 3:**
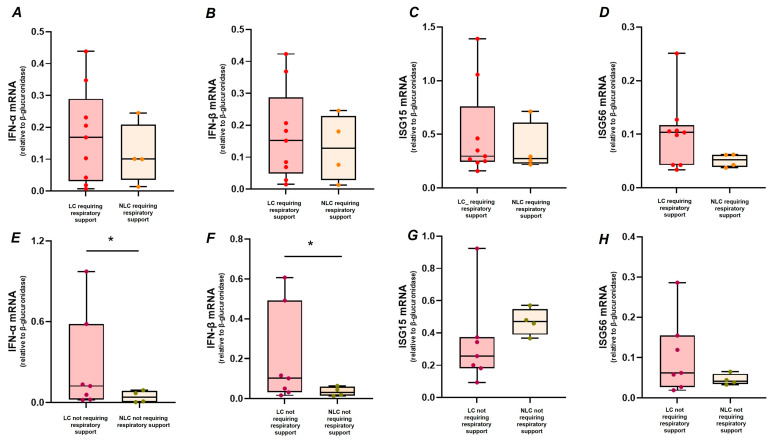
Gene expression levels of IFN-α (**A**,**E**), IFN-β (**B**,**F**), ISG15 (**C**,**G**), and ISG56 (**D**,**H**) in PBMC of LC (*n* = 9) and NLC (*n* = 4), (VM, HFNC, CPAP) during hospitalization, and LC (*n* = 7) and NLC (*n* = 4), who did not require respiratory support during hospitalization. Gene expression values were compared between LC and NLC who required respiratory support during hospitalization and between LC and NLC who did not require respiratory support during hospitalization by the Mann–Whitney U-test, * *p*  <  0.05.

No differences were observed in the respiratory support required during hospitalization (VM, HFNC, or CPAP) between LC and NLC. However, similar levels of IFN-α (Figure 3A), IFN-β (Figure 3B), and the IFN-stimulated genes, ISG15 (Figure 3C) and ISG56 (Figure 3D) mRNAs were observed between LC (*n* = 9) and NLC (*n* = 4) requiring respiratory support during hospitalization (*p* > 0.05, for all genes analysed). On the other hand, participants who did not require respiratory support and developed LC (*n* = 7) exhibited higher levels of IFN-α (Figure 3E, *p* = 0.033) and IFN-β (Figure 3F, *p* = 0.019) compared to NLC (*n* = 4) with the same clinical characteristics.

## 3. Discussion

The definition of LC continues to evolve as new data are collected, improving the understanding of this chronic condition. A wide range of symptoms (more than 200) and clinical findings have been identified, including persistent fatigue, sleep disorders, and neurological disturbances, which can have a broad spectrum of physical, social, and psychological consequences [1,2,3,4]. The risk of developing LC is increased by pre-existing chronic health problems, and although it affects individuals of all ages, people of working age, especially those older than 45, are at greatest risk [19,20]. In this context, a recent meta-analysis reported that LC has a noteworthy impact on working-related activities, highlighting the persistence of unresolved symptoms after many months from acute infection [21]. Accordingly, in this study, a group of individuals in working age still exhibited post-acute infection sequelae after more than one year from SARS-CoV-2 infection, including neurologic cognitive manifestations, such as difficulty in the activities of daily living, sensitivity to bright and noise, and disturbance of attention and memory. This emphasizes that, beyond the initial phase of the disease, these subsequent conditions represent a chronic health issue with substantial socio-economic impacts. Treatment of SARS-CoV-2 infection with antivirals has been hypothesized to reduce the risk of long-term sequelae, mitigating disease severity, or reducing viral load and risk of subsequent viral persistence [22]. However, the healthcare system still faces the challenge of identifying effective treatment options for these lasting symptoms, partly due to the lack of distinct host clinical and immunological factors that could indicate the persistence of disturbances and serve as targets for specific drugs.

IFN-I is a tonic innate immune signal that persists in a long-lasting stimulation determined by the host’s immune fitness and microbiome immunosurveillance [23,24]; in the context of SARS-CoV-2 infection, IFN-I has been shown to be dysregulated as a result of complex host–response interactions [11,25]; in addition, this activation might occur to sites proximal to the CNS, since SARS-CoV-2-infected brain endothelial cells upregulate IFN-I signaling with interferon-induced transmembrane protein 2 (IFITM2), thus promoting neuroinflammation and neurodegeneration [26]. Therefore, a lasting systemic inflammation may explain the long-term effects of COVID-19 observed among people of working age. A continuous increase in IFN-I is recognized as a key pathogenic driver of several interferonopathies, and in the context of PASC, an overlap of symptoms has been described [14]. Hattori et al. hypothesize that PASC occurs through a first phase triggered by residual SARS-CoV-2 viral activity, followed by a secondary long-term autoimmune phase [14].

Despite there being no differences between ‘clinically defined’ LC and NLC, a trend toward an increase in the transcript levels of the main IFN-I pathway components (IFN-α and IFN-β) was observed among LC participants, suggesting that these IFN-I subtypes may play a key role in the development of LC symptoms [27]. In fact, in patients with PASC, the uncontrolled IFN-I activation is associated with a higher frequency of the CD14^+^ monocyte subset expressing STAT2, a transcription factor that regulates IFN-I and III IFN immunity and the inflammatory response during and after SARS-CoV-2 infection [28]. From a clinical perspective, a persistent expression of IFNs and other key immunological markers [Interleukin-6 (IL-6), Tumor Necrosis Factor- α (TNF-α), Granulocyte-Macrophage Colony-Stimulating Factor (GM-CSF), Macrophage Colony-Stimulating Factor (M-CSF)], as well as other signs of systemic inflammation, might contribute to immune injuries to several tissues and organs, which are prevalent in PASC [29]. In line with this, recent findings have shown that overactivation of the IFN response occurs alongside complement dysregulation and coagulation alterations, resulting in an increase in tissue injury markers in LC patients one year after the acute episode [30,31,32]. In this context, after almost one year since acute infection, a trend towards a reduction in ISG15, a ubiquitin-like pleiotropic protein that acts as a post-translational modifier of host and viral proteins, might be due to persistent defects in IFN-I cascade; in accordance, several regulatory proteins induced by SARS-CoV-2 infection, including inhibitors of activated STAT proteins and IFN-stimulated suppressors of cytokine signaling, concurrently inactivate the JAK-STAT pathway, resulting in a reduced expression of ISG15, even after many months from acute infection [33].

The incidence of LC after acute infection ranges from 50 to 85% for unvaccinated people who were hospitalized [34,35], and the PHOSP-COVID study highlights that the severity of physical and mental health impairments was closely related to the recovery after a long hospital stay [36]. In our study, we found an association between the transcript levels of IFN-β and the length of hospitalization for more than 10 days during COVID-19 among LC individuals, as compared to NLC. Interestingly, IFN-β belongs to a set of optimal analytes of the acute inflammatory response that are strongly associated with LC, alongside Pentraxin-related protein 3 (PTX3), IFN-γ, IFN-λ2/3, and IL-6 [37], supporting the existence of a LC syndrome.

However, IFN-I subtypes might not be considered as independent factors for the development of respiratory symptoms recognized among the plethora of manifestations of PASC, in this study population. In fact, stratifying participants according to their need for respiratory support (VM, CPAP, or HFNC) during the acute infection, an association between higher levels of IFN-I subtypes and the development of LC was observed among participants with mild disease who did not require respiratory support. These data might suggest that, in cases where the innate immune response is altered, the subsequent adaptive immune response might be both timely and appropriate, thus helping in the control of SARS-CoV-2 infection and leading to the occurrence of a mild disease. However, one limitation of this analysis was that only three participants reported moderate shortness of breath or difficulty breathing during acute infection, thereby limiting the association between a failure to resolve innate responses and the development of LC, according to disease severity.

In addition, the variation in the rate of recovery from acute SARS-CoV-2 infection might be associated with modulation of IFNs and ISGs at the transcriptional level, thus explaining the persistence of symptoms associated with LC in individuals of working age, helping to pave the way to develop specific therapies and give some patients a solid diagnosis.

Nevertheless, the study has some limitations that should be discussed: (i) the present study should be considered as pilot research as the small sample size may have limited the statistical concerns about differences between LC and NLC. Since participants were enrolled when restrictive measures for COVID-19 pandemics were still active in Italy, as a result, some categories of patients encountered difficulties in accessing clinics and undergoing blood sampling and clinical assessments. Therefore, a larger study should be performed to extend these findings; (ii) the quantification of IFN-I and ISGs protein level through more sensitive assays should support our observation and help in better understanding the contribution of this pathways in the occurrence of post sequalae symptoms; however, it is well defined that most protein level dynamics were determined by mRNA level variations, and the latest technologies and refined mathematical modelling suggest that differences between protein levels are largely explained by changes in transcript concentrations. In addition, real-time PCR is a highly reproducible, reliable, not expensive, quantitative assay, performed with gene-specific probes, that allow us to analyze the expression of different cellular genes, also from residual sample, and add information to the existing knowledge; (iii) all participants had a mild/moderate disease and did not require ICU admission, thus precluding the association of LC and innate immunological features with severe COVID-19; (iv) the no-availability of gene expression data during acute infection and from a control group of healthy volunteers is due to timing of ethics approval and cohort setup during the first waves of pandemics, therefore it was not possible to assess whether the expression of these transcripts after one year since hospitalization is consistent with that occurring during acute SARS-CoV-2 and differed from those observed among participants with no history of virus exposure. However, a previous analysis highlighted an alteration of IFNs and ISGs expression among SARS-CoV-2-infected individuals, as compared to healthy individuals [38]; therefore, the main contribution of this analysis was to point out the role of these innate immunity pathways and provide a complete overview of immunological perturbances occurring from acute infection to post-acute sequelae.

In conclusion, these data suggest that the modulation of peripheral innate immune pathways one year after SARS-CoV-2 infection clearance might have important implications for our understanding of the immunopathological features of symptoms associated with LC. Our results might provide a significant support to other data from literature that revealed alterations in type I IFN, including IFN-α and IFN-β, among individuals with post-acute sequelae of COVID-19 (from children to adults) as compared to healthy controls and those recovered from an acute infection of SARS-CoV-2 without long COVID [14,27]. These alterations may have also implications for how individuals recovering from COVID-19 respond to SARS-CoV-2 vaccination or to other challenges faced after acute infection, considering that this persistent state of inflammation may also exacerbate other chronic conditions.

Focusing on people of working age, this study highlights the importance of addressing LC symptoms and accounting for long-term effects in specific populations, with the aim of developing an adequate approach for limiting the consequences on work performance. This study, therefore, provides new insights into the immunopathogenesis of LC in this age group and helps identify immunotherapies to mitigate impacts on well-being and work performance. A more structured approach could involve specialist consultations, precise diagnostic procedures, and rehabilitation services, as well as preventive measures, despite the increased healthcare costs [39].

## 4. Materials and Methods

### 4.1. Study Design and Participants

This study was designed as a cross-sectional study with a retrospective SARS-CoV-2 exposure assessment. All patients with confirmed previous SARS-CoV-2 infection hospitalized at the Department of Public Health and Infectious Diseases, Umberto I Hospital, Sapienza University of Rome (Italy), between March 2020 and November 2021, were considered candidates to be enrolled. Inclusion criteria were as follows: (i) male and female adults in working age, (ii) hospitalization for SARS-CoV-2 infection about one year prior to the enrolment for the present study, (iii) no documented history or compatible symptoms of SARS-CoV-2 infection from hospital discharge to the time of enrollment, and (iv) a negative SARS-CoV-2 test during the study visit day. Exclusion criteria were pregnancy status, contraindications for blood sampling, major medical illness, any major psychiatric or neurological illnesses, and any substance addiction or significant chronic consumption and the relative medications. All participants were examined to ascertain their physical, clinical, and cognitive status by brief interviews assessing medical history, medication use, parental psychopathology, demographics, psychiatric symptoms, alcohol and drug use, and general cognitive status. Peripheral blood samples were obtained from a subgroup of previously SARS-CoV-2-infected participants (*n* = 24). All patients gave informed consent to participate in the study and to have their samples collected. The following variables were collected from electronic medical records: age, gender, date of admission and discharge from the hospital, length of hospital stay, comorbidities, C-reactive protein, and type of respiratory support during hospitalization. As recommended by the Italian Society of Infectious and Tropical Diseases (abbreviated as SIMIT), patients received therapeutic regimens including remdesivir and low-molecular-weight heparin for prophylaxis of deep vein thrombosis. The study was approved by the Ethics Committee and was conducted in compliance with the Declaration of Helsinki, the Good Clinical Practice guidelines, and local regulatory requirements (protocol code 6484/2021, date of approval 29/09/2021).

### 4.2. Diagnosis of Long COVID

LC were defined as cases in which participants reported at least two symptoms, according to the De Paul Symptom Questionnaire [40], developed and successfully tested to measure chronic fatigue syndrome (CFS) symptomatology. It can accurately differentiate between individuals with CFS and healthy controls or subjects with other chronic illnesses [41]. Considering that, to date, validated and specific tools for the assessment of LC symptoms are still lacking, we administered the DePaul Symptom Questionnaire 54 questions to assess several conditions that are widely acknowledged as post-acute sequelae.

The investigation of LC symptoms was performed by collecting clinically relevant information from the hospital discharge to the time of enrolment, as previously reported [42]. Briefly, the investigated symptoms included post exertional malaise (PEM) (Fatigue/extreme tiredness, dead, heavy feeling after starting to exercise, Mentally tired after the slightest effort, Physically tired after the minimum exercise, Physically drained or sick after mild activity), sleep disorders (feeling unrefreshed after waking up in the morning, need to nap daily, problems in falling asleep, problems staying asleep), musculoskeletal symptoms (muscle twitches, muscle weakness), neurological and cognitive manifestations (sensitivity to noise, sensitivity to bright lights, problems remembering things, difficulty paying attention for a long period, difficulty finding the right word to say or expressing thoughts, difficulty understanding things, unable to focus vision and/or attention, loss of depth perception, slowness of thought, absent-mindedness or forgetfulness). These symptoms were attributed to LC syndrome only if (1) they appeared after the original SARS-CoV-2infection, (2) lasted for at least two months after the ascertained negativization of swab, and (3) could not be explained by an alternative diagnosis.

### 4.3. Anti-IFN-I Autoantibodies Detection

Plasma samples from all study participants were tested for NAB against IFN-α (IFN-α2a, Intron; Schering-Plough, Kenilworth, NJ, USA) and IFN-β (Rebif, Serono, Geneva, Switzerland), in a bioassay based on IFN-induced inhibition of the encephalomyocarditis (EMC) virus cytopathic effect on human lung carcinoma epithelial cells (A549), as previously described [11]. Titers were calculated using Kawade’s method, and the titers were expressed as TRU/mL, where one TRU is the plasma dilution capable of reducing the IFN titer from 10 to 1 IU/mL.

### 4.4. Peripheral Blood Mononuclear Cells Isolation and Real-Time PCR Assay

Levels of mRNAs for IFN-I (IFN-α2, IFN-β) and selected ISGs (ISG15, ISG56) were assessed on Peripheral Blood Mononuclear Cells (PBMC) by quantitative RT(real-time) PCR assays, using the LightCycler-480 (Roche). PBMC were isolated from fresh peripheral blood samples (20 mL) collected from a subgroup of previously SARS-CoV-2 infected study participants (*n* = 24) and processed by Ficoll-Hypaque density gradient centrifugation (Lympholyte, Cedarlane Labs, Burlington, ON, Canada). Dried pellets were stored at −80 °C for RNA extraction. Briefly, total RNA was extracted from PBMC pellets using a commercially available RNA purification assay (ZymoBIOMICS RNA Miniprep Kit-Zymo Research, Irvine, CA, USA) and reverse transcribed using the High-Capacity cDNA Reverse Transcription Kit (Applied Biosystems, Waltham, MA, USA), according to the manufacturer’s protocol. RNA quality was measured using NanoDrop spectrophotometers, loading 2 µL of sample; RNA concentration was determined by reading absorbance at 260 nm and 280 nm. All primers and probes were added to the Probes Master Mix (Roche, Basel, Switzerland) at 500 and 250 nm, respectively, in a final volume of 20 μL. The housekeeping gene β-glucuronidase (GUS) was used as an internal control. Gene expression levels were calculated using the comparative cycle threshold value (Ct) method (2^−ΔCt^). All RT-PCR reactions were performed in duplicate. The primer and probe sequences targeting IFN-α2, IFN-β, ISG15, and ISG56 have been previously reported [11,25,27,43].

### 4.5. Statistical Analysis

Study participants’ data were expressed as mean (±SD), median/IQR (interquartile range), or as frequency (percentage). The demographic and clinical characteristics of previously SARS-CoV-2-infected LC and NLC participants were compared using Student’s t and χ^2^ tests. Differences in IFN-α, IFN-β, ISG15, and ISG56 mRNA levels between LC and NLC previously SARS-CoV-2-infected participants were analysed using the Mann–Whitney U-test, performed with SPSS v.27 for Windows. Then, three multivariable linear regression models were constructed to assess the impact of selected factors on the expression levels of IFN-α mRNA (Model 1), IFN-β mRNA (Model 2), and ISG56 mRNA (Model 3). A model for ISG15 mRNA could not be developed due to limitations in data distribution. To improve model robustness and fulfil linear regression assumptions, the dependent variable was log-transformed when appropriate (models 1 and 3). Independent variables were selected based on expert opinion and/or statistical relevance in univariate analyses (*p* < 0.25). All final models included the following covariates: LC status (0 = no; 1 = yes), CCI at the time of hospitalization (0 = <2; 1 = ≥2), length of hospitalization (0 = <10 days; 1 = ≥10 days), and need for respiratory support during hospitalization (0 = no; 1 = yes). Multivariable linear regressions were conducted using StataNow version 18.5 (StataCorp LLC, College Station, TX, USA). A *p*-value less than 0.05 was considered statistically significant.

## Figures and Tables

**Table 1:** Demographic and clinical parameters of the study population.

Parameters *	Previous SARS-CoV-2 Infection (*n* = 34) **	LC (*n* = 26) (A)	NLC (*n* = 8) (B)	*p*-Value A vs. B
Age (years)	59 (±6)	58 (±5)	60 (±6)	0.395
Sex assigned at birth (male/female)	20/14	15/11	6/2	NA
Days between last SARS-CoV-2 swab and blood sampling (days)	481 (413–551)	483 (379–547)	486 (472–590)	0.262
Comorbidities (n, %) ***	9 (26.5%)	7 (27%)	2 (25%)	0.912
Length of hospitalization (days)	10 (6–17)	10 (7–18)	8 (5–14)	0.788
ICU admission [n (%)]	0 (0%)	0 (0%)	0 (0%)	1.00
C-reactive protein (mg/dL)	2.8 (0.99–5.25)	2.69 (1.2–5.41)	2.49 (0.76–7.78)	0.917
Respiratory support during hospitalization				
Venti-mask [n (%)]	14 (41%)	12 (46%)	2 (25%)	0.298
HFNC [n (%)]	5 (14.7%)	3 (11.5%)	2 (25%)	0.352
CPAP [n (%)]	9 (26%)	6 (23%)	3 (37.5%)	0.422

* Data were expressed as mean (±standard deviation), median (25° percentile–75° percentile), and number (percentages). ** All patients were treated with the best available therapeutic regimens (antivirals, azithromycin, or steroids) during acute infection. Data regarding length of hospitalization, ICU admission, C-reactive protein, as well as respiratory support during hospitalization, have been collected during acute SARS-CoV-2 infection. *** Comorbidities included Type 1 diabetes mellitus, Type 2 diabetes mellitus, hypertension, immune thrombocytopenia, asthma, previous LNH, and hypercholesterolemia. Statistical analysis was performed by using Student’s *t* and χ2 tests. LC: long COVID; NLC: non-long COVID; ICU: Intensive Care Unit; HFNC: High Flow Nasal Cannula; CPAP: Continuous Positive Airway Pressure.

**Table 2:** Post-COVID symptoms among LC and NLC participants.

Symptoms *	LC n (%)	NLC n (%)
Fatigue **	8 (50%)	1 (12.5)
Sleep disorders ***	14 (87.5)	1 (12.5)
Musculoskeletal symptoms ****	3 (18.8)	0 (0)
Neurocognitive manifestations *****	14 (87.5)	3 (37.5)

* Data were collected according to the De Paul Symptom Questionnaire (https://condor.depaul.edu/ljason/cfs/measures.html (accessed on 10 November 2021). ** Fatigue/extreme tiredness; dead, heavy feeling after starting to exercise; mentally tired after the slightest effort; physically tired after the minimum exercise; physically drained or sick after mild activity. *** Feeling unrefreshed after waking up in the morning; need to nap daily; problems in falling asleep; problems staying asleep. **** Muscle twitches; muscle weakness. ***** Sensitivity to noise; sensitivity to bright lights; problems remembering things; difficulty paying attention for a long period; difficulty finding the right word to say or expressing thoughts; difficulty understanding things; unable to focus vision and/or attention; loss of depth perception; slowness of thought; absent-mindedness or forgetfulness.

**Table 3:** Linear regression models predicting levels of IFN-α mRNA, IFN-β mRNA, and ISG56 mRNA.

	IFN-α mRNA *			IFN-β mRNA			ISG56 mRNA *		
	aβ	95% CI	*p*-Value	aβ	95% CI	*p*-Value	aβ	95% CI	*p*-Value
Long COVID (yes)	0.89	−0.34 to 2.13	0.148	0.10	−0.02 to 0.21	0.090	0.55	0.11 to 0.99	**0.016**
CCI (≥2)	−1.54	−2.80 to −0.27	**0.020**	−0.18	−0.29 to −0.07	**0.002**	−0.62	−1.18 to −0.05	**0.034**
Days of hospitalization (≥10)	−0.08	−1.44 to 1.28	0.903	0.06	−0.07 to 0.19	0.360	0.51	0.08 to 0.94	**0.022**
Respiratory support (yes)	0.25	−4.39 to −1.25	0.698	−0.03	−0.18 to 0.12	0.677	−0.16	−0.67 to 0.35	0.522

Abbreviation: aβ, adjusted regression coefficients; CI, confidence interval; CCI, Charlson Comorbidity Index. * Log-transformed to satisfy regression assumptions. Significant values (*p* < 0.05) are shown in bold.

## Data Availability

The datasets analysed for this study can be provided by the corresponding author upon reasonable request.

## References

[1] Nalbandian A., Sehgal K., Gupta A., Madhavan M.V., McGroder C., Stevens J.S., Cook J.R., Nordvig A.S., Shalev D., Sehrawat T.S. (2021). Post-acute COVID-19 syndrome. Nat. Med..

[2] Sudre C.H., Murray B., Varsavsky T., Graham M.S., Penfold R.S., Bowyer R.C., Pujol J.C., Klaser K., Antonelli M., Canas L.S. (2021). Attributes and predictors of long COVID. Nat. Med..

[3] Davis H.E., Assaf G.S., McCorkell L., Wei H., Low R.J., Re’em Y., Redfield S., Austin J.P., Akrami A. (2021). Characterizing long COVID in an international cohort: 7 months of symptoms and their impact. EClinicalMedicine.

[4] Huang L., Yao Q., Gu X., Wang Q., Ren L., Wang Y., Hu P., Guo L., Liu M., Xu J. (2021). 1-year outcomes in hospital survivors with COVID-19: A longitudinal cohort study. Lancet.

[5] Lazear H.M., Schoggins J.W., Diamond M.S. (2019). Shared and Distinct Functions of Type I and Type III Interferons. Immunity.

[6] Blanco-Melo D., Nilsson-Payant B.E., Liu W.C., Uhl S., Hoagland D., Møller R., Jordan T.X., Oishi K., Panis M., Sachs D. (2020). Imbalanced Host Response to SARS-CoV-2 Drives Development of COVID-19. Cell.

[7] Hadjadj J., Yatim N., Barnabei L., Corneau A., Boussier J., Smith N., Péré H., Charbit B., Bondet V., Chenevier-Gobeaux C. (2020). Impaired type I interferon activity and inflammatory responses in severe COVID-19 patients. Science.

[8] Park A., Iwasaki A. (2020). Type I and Type III Interferons-Induction, Signaling, Evasion, and Application to Combat COVID-19. Cell Host Microbe..

[9] Lei X., Dong X., Ma R., Wang W., Xiao X., Tian Z., Wang C., Wang Y., Li L., Ren L. (2020). Activation and evasion of type I interferon responses by SARS-CoV-2. Nat. Commun..

[10] Song L., Wang D., Abbas G., Li M., Cui M., Wang J., Lin Z., Zhang X.E. (2023). The main protease of SARS-CoV-2 cleaves histone deacetylases and DCP1A, attenuating the immune defense of the interferon-stimulated genes. J. Biol. Chem..

[11] Frasca F., Scordio M., Santinelli L., Gabriele L., Gandini O., Criniti A., Pierangeli A., Angeloni A., Mastroianni C.M., d’Ettorre G. (2022). Anti-IFN-α/-ω neutralizing antibodies from COVID-19 patients correlate with downregulation of IFN response and laboratory biomarkers of disease severity. Eur. J. Immunol..

[12] Patterson B.K., Guevara-Coto J., Yogendra R., Francisco E.B., Long E., Pise A., Rodrigues H., Parikh P., Mora J., Mora-Rodríguez R.A. (2021). Immune-Based Prediction of COVID-19 Severity and Chronicity Decoded Using Machine Learning. Front. Immunol..

[13] Su Y., Yuan D., Chen D.G., Ng R.H., Wang K., Choi J., Li S., Hong S., Zhang R., Xie J. (2022). Multiple early factors anticipate post-acute COVID-19 sequelae. Cell.

[14] Hattori F., Nishiyama J., Hasuo H. (2025). Correlation of interferons and autoimmune aspects in long COVID-19 patients. Int. Immunol..

[15] Deer R.R., Rock M.A., Vasilevsky N., Carmody L., Rando H., Anzalone A.J., Basson M.D., Bennett T.D., Bergquist T., Boudreau E.A. (2021). Characterizing Long COVID: Deep Phenotype of a Complex Condition. EBioMedicine.

[16] Van Herck M., Goërtz Y.M., Houben-Wilke S., Machado F.V., Meys R., Delbressine J.M., Vaes A.W., Burtin C., Posthuma R., Franssen F.M. (2021). Severe Fatigue in Long COVID: Web-Based Quantitative Follow-up Study in Members of Online Long COVID Support Groups. J. Med. Internet. Res..

[17] Ayoubkhani D., Zaccardi F., Pouwels K.B., Walker A.S., Houston D., Alwan N.A., Martin J., Khunti K., Nafilyan V. (2024). Employment outcomes of people with Long Covid symptoms: Community-based cohort study. Eur. J. Public Health.

[18] Mortaz E., Tabarsi P., Varahram M., Folkerts G., Adcock I.M. (2020). The Immune Response and Immunopathology of COVID-19. Front. Immunol..

[19] Davis H.E., McCorkell L., Vogel J.M., Topol E.J. (2023). Long COVID: Major findings, mechanisms and recommendations. Nat. Rev. Microbiol..

[20] Thompson E.J., Williams D.M., Walker A.J., Mitchell R.E., Niedzwiedz C.L., Yang T.C., Huggins C.F., Kwong A.S., Silverwood R.J., Di Gessa G. (2022). Long COVID burden and risk factors in 10 UK longitudinal studies and electronic health records. Nat. Commun..

[21] Rahmati M., Udeh R., Kang J., Dolja-Gore X., McEvoy M., Kazemi A., Soysal P., Smith L., Kenna T., Fond G. (2025). Long-Term Sequelae of COVID-19: A Systematic Review and Meta-Analysis of Symptoms 3 Years Post-SARS-CoV-2 Infection. J. Med. Virol..

[22] Al-Aly Z. (2025). SARS-CoV-2 antivirals and post-COVID-19 condition. Lancet Infect. Dis..

[23] Lukhele S., Boukhaled G.M., Brooks D.G. (2019). Type I interferon signaling, regulation and gene stimulation in chronic virus infection. Semin. Immunol..

[24] Zheng D., Liwinski T., Elinav E. (2020). Interaction between microbiota and immunity in health and disease. Cell Res..

[25] Maddaloni L., Santinelli L., Bugani G., Cacciola E.G., Lazzaro A., Lofaro C.M., Caiazzo S., Frasca F., Fracella M., Ajassa C. (2023). Differential expression of Type I interferon and inflammatory genes in SARS-CoV-2-infected patients treated with monoclonal antibodies. Immun. Inflamm. Dis..

[26] Yang A.C., Kern F., Losada P.M., Agam M.R., Maat C.A., Schmartz G.P., Fehlmann T., Stein J.A., Schaum N., Lee D.P. (2021). Dysregulation of brain and choroid plexus cell types in severe COVID-19. Nature.

[27] Fracella M., Mancino E., Nenna R., Virgillito C., Frasca F., D’Auria A., Sorrentino L., Petrarca L., La Regina D., Matera L. (2024). Age-related transcript changes in type I interferon signaling in children and adolescents with long COVID. Eur. J. Immunol..

[28] Xu D., Qin X. (2024). Type I Interferonopathy among Non-Elderly Female Patients with Post-Acute Sequelae of COVID-19. Viruses.

[29] Gusev E., Sarapultsev A. (2024). Exploring the Pathophysiology of Long COVID: The Central Role of Low-Grade Inflammation and Multisystem Involvement. Int. J. Mol. Sci..

[30] Cervia-Hasler C., Brüningk S.C., Hoch T., Fan B., Muzio G., Thompson R.C., Ceglarek L., Meledin R., Westermann P., Emmenegger M. (2024). Persistent complement dysregulation with signs of thromboinflammation in active Long Covid. Science.

[31] Tripathi A., Whitehead C., Surrao K., Pillai A., Madeshiya A., Li Y., Khodadadi H., Ahmed A.O., Turecki G., Baban B. (2021). Type 1 interferon mediates chronic stress-induced neuroinflammation and behavioral deficits via complement component 3-dependent pathway. Mol. Psychiatry.

[32] Jodele S., Medvedovic M., Luebbering N., Chen J., Dandoy C.E., Laskin B.L., Davies S.M. (2020). Interferon-complement loop in transplant-associated thrombotic microangiopathy. Blood Adv..

[33] Sarkar L., Liu G., Gack M.U. (2023). ISG15: Its roles in SARS-CoV-2 and other viral infections. Trends Microbiol..

[34] Bowe B., Xie Y., Al-Aly Z. (2023). Postacute sequelae of COVID-19 at 2 years. Nat. Med..

[35] Pavli A., Theodoridou M., Maltezou H.C. (2021). Post-COVID Syndrome: Incidence, Clinical Spectrum, and Challenges for Primary Healthcare Professionals. Arch. Med. Res..

[36] Evans R.A., McAuley H., Harrison E.M., Shikotra A., Singapuri A., Sereno M., Elneima O., Docherty A.B., Lone N.I., Leavy O.C. (2021). Physical, cognitive, and mental health impacts of COVID-19 after hospitalisation (PHOSP-COVID): A UK multicentre, prospective cohort study. Lancet Respir. Med..

[37] Phetsouphanh C., Darley D.R., Wilson D.B., Howe A., Munier C.M.L., Patel S.K., Juno J.A., Burrell L.M., Kent S.J., Dore G.J. (2022). Immunological dysfunction persists for 8 months following initial mild-to-moderate SARS-CoV-2 infection. Nat. Immunol..

[38] Pierangeli A., Gentile M., Oliveto G., Frasca F., Sorrentino L., Matera L., Nenna R., Viscido A., Fracella M., Petrarca L. (2022). Comparison by Age of the Local Interferon Response to SARS-CoV-2 Suggests a Role for IFN-ε and -ω. Front. Immunol..

[39] Sweis J.J.G., Alnaimat F., Esparza V., Prasad S., Azam A., Modi Z., Al-Awqati M., Jetanalin P., Sweis N.J., Ascoli C. (2024). From Acute Infection to Prolonged Health Consequences: Understanding Health Disparities and Economic Implications in Long COVID Worldwide. Int. J. Environ. Res. Public Health.

[40] De Paul University https://condor.depaul.edu/ljason/cfs/measures.html.

[41] Jason L.A., Evans M., Porter N., Brown M., Brown A., Hunnell J., Anderson V., Lerch A., De Meirleir K., Friedberg F. (2010). The Development of a Revised Canadian Myalgic Encephalomyelitis Chronic Fatigue Syndrome Case Definition. Am. J. Biochem. Biotechnol..

[42] Babiloni C., Cacciola E.G., Tucci F., Vassalini P., Chilovi A., Jakhar D., Musat A.M., Salvatore M., Soricelli A., Stocchi F. (2024). Resting-state EEG rhythms are abnormal in post COVID-19 patients with brain fog without cognitive and affective disorders. Clin. Neurophysiol..

[43] Santinelli L., De Girolamo G., Borrazzo C., Vassalini P., Pinacchio C., Cavallari E.N., Statzu M., Frasca F., Scordio M., Bitossi C. (2021). Alteration of type I interferon response is associated with subclinical atherosclerosis in virologically suppressed HIV-1-infected male patients. J. Med. Virol..

